# Supplementary material for: Modeling glioblastoma heterogeneity as a dynamic network of cell states
Source: Mol Syst Biol. 2021 Sep 16;17(9):e10105. doi: 10.15252/msb.202010105 (PMC8444284; doi:10.15252/msb.202010105)
Supplement: Supplementary file 5 — Source Data for Figure 3 [file MSB-17-e10105-s001.zip › Figure3A_sourcedata/GSEA_3065/hallmarks_state2.GseaPreranked.1623416269049/HALLMARK_E2F_TARGETS.html]

Details for gene set HALLMARK\_E2F\_TARGETS[GSEA]

|  || Dataset | state2 |
| Phenotype | NoPhenotypeAvailable |
| Upregulated in class | na\_pos |
| GeneSet | HALLMARK\_E2F\_TARGETS |
| Enrichment Score (ES) | 0.8224735 |
| Normalized Enrichment Score (NES) | 2.9509897 |
| Nominal p-value | 0.0 |
| FDR q-value | 0.0 |
| FWER p-Value | 0.0 |
Table: GSEA Results Summary

  

Fig 1: Enrichment plot: HALLMARK\_E2F\_TARGETS      
 Profile of the Running ES Score & Positions of GeneSet Members on the Rank Ordered List

  

| PROBE | GENE SYMBOL | GENE\_TITLE | RANK IN GENE LIST | RANK METRIC SCORE | RUNNING ES | CORE ENRICHMENT || 1 | HMGB2 |  |  | 4 | 0.823 | 0.0169 | Yes |
| 2 | UBE2T |  |  | 12 | 0.643 | 0.0297 | Yes |
| 3 | MCM7 |  |  | 13 | 0.626 | 0.0428 | Yes |
| 4 | MYBL2 |  |  | 14 | 0.600 | 0.0555 | Yes |
| 5 | TOP2A |  |  | 15 | 0.596 | 0.0680 | Yes |
| 6 | PCNA |  |  | 16 | 0.595 | 0.0805 | Yes |
| 7 | MKI67 |  |  | 17 | 0.590 | 0.0930 | Yes |
| 8 | CDK1 |  |  | 18 | 0.577 | 0.1051 | Yes |
| 9 | UBE2S |  |  | 19 | 0.574 | 0.1172 | Yes |
| 10 | MAD2L1 |  |  | 20 | 0.563 | 0.1290 | Yes |
| 11 | ATAD2 |  |  | 21 | 0.563 | 0.1409 | Yes |
| 12 | RRM2 |  |  | 22 | 0.556 | 0.1526 | Yes |
| 13 | CKS1B |  |  | 23 | 0.554 | 0.1642 | Yes |
| 14 | DUT |  |  | 24 | 0.552 | 0.1759 | Yes |
| 15 | CKS2 |  |  | 28 | 0.532 | 0.1868 | Yes |
| 16 | DEK |  |  | 31 | 0.526 | 0.1976 | Yes |
| 17 | STMN1 |  |  | 33 | 0.520 | 0.2085 | Yes |
| 18 | RPA3 |  |  | 36 | 0.511 | 0.2190 | Yes |
| 19 | ORC6 |  |  | 40 | 0.493 | 0.2291 | Yes |
| 20 | SMC4 |  |  | 42 | 0.489 | 0.2392 | Yes |
| 21 | TMPO |  |  | 43 | 0.489 | 0.2495 | Yes |
| 22 | KPNA2 |  |  | 50 | 0.472 | 0.2588 | Yes |
| 23 | MCM3 |  |  | 51 | 0.464 | 0.2686 | Yes |
| 24 | RFC2 |  |  | 53 | 0.460 | 0.2782 | Yes |
| 25 | CENPM |  |  | 54 | 0.460 | 0.2878 | Yes |
| 26 | TK1 |  |  | 56 | 0.456 | 0.2973 | Yes |
| 27 | BIRC5 |  |  | 61 | 0.443 | 0.3062 | Yes |
| 28 | PTTG1 |  |  | 63 | 0.439 | 0.3153 | Yes |
| 29 | RANBP1 |  |  | 65 | 0.430 | 0.3243 | Yes |
| 30 | LMNB1 |  |  | 69 | 0.417 | 0.3327 | Yes |
| 31 | EZH2 |  |  | 70 | 0.417 | 0.3415 | Yes |
| 32 | ASF1B |  |  | 76 | 0.407 | 0.3495 | Yes |
| 33 | MCM4 |  |  | 79 | 0.399 | 0.3577 | Yes |
| 34 | TUBB |  |  | 81 | 0.397 | 0.3660 | Yes |
| 35 | MCM5 |  |  | 83 | 0.394 | 0.3741 | Yes |
| 36 | KIF4A |  |  | 84 | 0.393 | 0.3824 | Yes |
| 37 | HNRNPD |  |  | 88 | 0.390 | 0.3903 | Yes |
| 38 | RAD51AP1 |  |  | 90 | 0.388 | 0.3983 | Yes |
| 39 | BRCA1 |  |  | 92 | 0.381 | 0.4063 | Yes |
| 40 | CDKN3 |  |  | 97 | 0.376 | 0.4137 | Yes |
| 41 | RAN |  |  | 98 | 0.375 | 0.4216 | Yes |
| 42 | SPC25 |  |  | 104 | 0.372 | 0.4289 | Yes |
| 43 | CENPE |  |  | 105 | 0.372 | 0.4367 | Yes |
| 44 | SLBP |  |  | 112 | 0.357 | 0.4436 | Yes |
| 45 | POP7 |  |  | 119 | 0.347 | 0.4503 | Yes |
| 46 | POLD3 |  |  | 120 | 0.347 | 0.4576 | Yes |
| 47 | DNMT1 |  |  | 121 | 0.345 | 0.4648 | Yes |
| 48 | CDKN2C |  |  | 123 | 0.341 | 0.4719 | Yes |
| 49 | TACC3 |  |  | 130 | 0.334 | 0.4783 | Yes |
| 50 | CDK4 |  |  | 131 | 0.333 | 0.4853 | Yes |
| 51 | SNRPB |  |  | 132 | 0.332 | 0.4923 | Yes |
| 52 | MELK |  |  | 133 | 0.332 | 0.4993 | Yes |
| 53 | ANP32E |  |  | 136 | 0.330 | 0.5060 | Yes |
| 54 | PA2G4 |  |  | 137 | 0.329 | 0.5130 | Yes |
| 55 | USP1 |  |  | 138 | 0.329 | 0.5199 | Yes |
| 56 | RAD21 |  |  | 147 | 0.316 | 0.5257 | Yes |
| 57 | MCM6 |  |  | 149 | 0.311 | 0.5321 | Yes |
| 58 | KIF22 |  |  | 154 | 0.306 | 0.5381 | Yes |
| 59 | NASP |  |  | 156 | 0.306 | 0.5445 | Yes |
| 60 | PSMC3IP |  |  | 159 | 0.305 | 0.5507 | Yes |
| 61 | CDCA3 |  |  | 160 | 0.304 | 0.5571 | Yes |
| 62 | HELLS |  |  | 161 | 0.304 | 0.5635 | Yes |
| 63 | AURKB |  |  | 163 | 0.303 | 0.5698 | Yes |
| 64 | DCTPP1 |  |  | 172 | 0.296 | 0.5751 | Yes |
| 65 | PSIP1 |  |  | 175 | 0.294 | 0.5811 | Yes |
| 66 | DDX39A |  |  | 183 | 0.293 | 0.5865 | Yes |
| 67 | BARD1 |  |  | 185 | 0.292 | 0.5925 | Yes |
| 68 | PAICS |  |  | 187 | 0.291 | 0.5985 | Yes |
| 69 | CCNB2 |  |  | 190 | 0.286 | 0.6044 | Yes |
| 70 | CDC20 |  |  | 193 | 0.284 | 0.6101 | Yes |
| 71 | DEPDC1 |  |  | 196 | 0.283 | 0.6159 | Yes |
| 72 | SMC3 |  |  | 197 | 0.283 | 0.6218 | Yes |
| 73 | HMGB3 |  |  | 200 | 0.282 | 0.6276 | Yes |
| 74 | SRSF2 |  |  | 213 | 0.275 | 0.6320 | Yes |
| 75 | NME1 |  |  | 214 | 0.274 | 0.6378 | Yes |
| 76 | CHEK1 |  |  | 224 | 0.266 | 0.6424 | Yes |
| 77 | CBX5 |  |  | 225 | 0.265 | 0.6480 | Yes |
| 78 | MCM2 |  |  | 228 | 0.262 | 0.6533 | Yes |
| 79 | JPT1 |  |  | 236 | 0.260 | 0.6580 | Yes |
| 80 | SMC1A |  |  | 249 | 0.253 | 0.6621 | Yes |
| 81 | KIF2C |  |  | 252 | 0.251 | 0.6671 | Yes |
| 82 | SPC24 |  |  | 256 | 0.248 | 0.6720 | Yes |
| 83 | LIG1 |  |  | 259 | 0.246 | 0.6770 | Yes |
| 84 | RAD51C |  |  | 260 | 0.246 | 0.6822 | Yes |
| 85 | TUBG1 |  |  | 262 | 0.246 | 0.6872 | Yes |
| 86 | MSH2 |  |  | 266 | 0.244 | 0.6921 | Yes |
| 87 | TIMELESS |  |  | 272 | 0.243 | 0.6966 | Yes |
| 88 | TRIP13 |  |  | 286 | 0.237 | 0.7002 | Yes |
| 89 | TCF19 |  |  | 318 | 0.226 | 0.7016 | Yes |
| 90 | SSRP1 |  |  | 319 | 0.226 | 0.7064 | Yes |
| 91 | DIAPH3 |  |  | 321 | 0.226 | 0.7110 | Yes |
| 92 | MXD3 |  |  | 328 | 0.224 | 0.7151 | Yes |
| 93 | POLD2 |  |  | 330 | 0.223 | 0.7197 | Yes |
| 94 | RACGAP1 |  |  | 333 | 0.222 | 0.7241 | Yes |
| 95 | HMMR |  |  | 358 | 0.215 | 0.7261 | Yes |
| 96 | PLK1 |  |  | 364 | 0.213 | 0.7300 | Yes |
| 97 | POLA2 |  |  | 366 | 0.212 | 0.7344 | Yes |
| 98 | RPA2 |  |  | 367 | 0.212 | 0.7388 | Yes |
| 99 | DLGAP5 |  |  | 371 | 0.211 | 0.7430 | Yes |
| 100 | AURKA |  |  | 375 | 0.210 | 0.7471 | Yes |
| 101 | NAP1L1 |  |  | 387 | 0.207 | 0.7502 | Yes |
| 102 | CSE1L |  |  | 391 | 0.204 | 0.7542 | Yes |
| 103 | DSCC1 |  |  | 395 | 0.202 | 0.7581 | Yes |
| 104 | NOP56 |  |  | 396 | 0.201 | 0.7623 | Yes |
| 105 | UNG |  |  | 398 | 0.200 | 0.7665 | Yes |
| 106 | BRCA2 |  |  | 427 | 0.193 | 0.7675 | Yes |
| 107 | NCAPD2 |  |  | 434 | 0.193 | 0.7709 | Yes |
| 108 | SPAG5 |  |  | 439 | 0.192 | 0.7745 | Yes |
| 109 | TIPIN |  |  | 440 | 0.192 | 0.7785 | Yes |
| 110 | EXOSC8 |  |  | 456 | 0.189 | 0.7809 | Yes |
| 111 | UBR7 |  |  | 458 | 0.189 | 0.7848 | Yes |
| 112 | DCK |  |  | 465 | 0.187 | 0.7881 | Yes |
| 113 | GINS1 |  |  | 470 | 0.185 | 0.7915 | Yes |
| 114 | SYNCRIP |  |  | 503 | 0.180 | 0.7919 | Yes |
| 115 | TRA2B |  |  | 515 | 0.178 | 0.7944 | Yes |
| 116 | CDC25B |  |  | 519 | 0.178 | 0.7978 | Yes |
| 117 | BUB1B |  |  | 572 | 0.168 | 0.7957 | Yes |
| 118 | CDC25A |  |  | 578 | 0.167 | 0.7987 | Yes |
| 119 | POLE4 |  |  | 597 | 0.164 | 0.8002 | Yes |
| 120 | RFC3 |  |  | 617 | 0.161 | 0.8015 | Yes |
| 121 | CDCA8 |  |  | 634 | 0.158 | 0.8031 | Yes |
| 122 | E2F8 |  |  | 638 | 0.158 | 0.8061 | Yes |
| 123 | XRCC6 |  |  | 641 | 0.158 | 0.8092 | Yes |
| 124 | RFC1 |  |  | 655 | 0.156 | 0.8111 | Yes |
| 125 | PRIM2 |  |  | 667 | 0.154 | 0.8131 | Yes |
| 126 | RPA1 |  |  | 687 | 0.151 | 0.8143 | Yes |
| 127 | CTCF |  |  | 691 | 0.151 | 0.8171 | Yes |
| 128 | RBBP7 |  |  | 706 | 0.150 | 0.8188 | Yes |
| 129 | GINS4 |  |  | 727 | 0.146 | 0.8197 | Yes |
| 130 | PHF5A |  |  | 782 | 0.141 | 0.8168 | Yes |
| 131 | RAD1 |  |  | 789 | 0.139 | 0.8191 | Yes |
| 132 | MMS22L |  |  | 799 | 0.138 | 0.8210 | Yes |
| 133 | LYAR |  |  | 865 | 0.131 | 0.8168 | Yes |
| 134 | KIF18B |  |  | 879 | 0.130 | 0.8181 | Yes |
| 135 | CCNE1 |  |  | 888 | 0.129 | 0.8199 | Yes |
| 136 | DONSON |  |  | 929 | 0.125 | 0.8183 | Yes |
| 137 | AK2 |  |  | 948 | 0.124 | 0.8189 | Yes |
| 138 | PLK4 |  |  | 986 | 0.121 | 0.8175 | Yes |
| 139 | ILF3 |  |  | 1004 | 0.120 | 0.8181 | Yes |
| 140 | MTHFD2 |  |  | 1014 | 0.118 | 0.8197 | Yes |
| 141 | IPO7 |  |  | 1035 | 0.117 | 0.8199 | Yes |
| 142 | EIF2S1 |  |  | 1051 | 0.115 | 0.8207 | Yes |
| 143 | NOLC1 |  |  | 1104 | 0.111 | 0.8175 | Yes |
| 144 | MRE11 |  |  | 1106 | 0.111 | 0.8197 | Yes |
| 145 | SHMT1 |  |  | 1126 | 0.110 | 0.8200 | Yes |
| 146 | NAA38 |  |  | 1140 | 0.109 | 0.8208 | Yes |
| 147 | NUP205 |  |  | 1147 | 0.108 | 0.8225 | Yes |
| 148 | CTPS1 |  |  | 1223 | 0.103 | 0.8165 | No |
| 149 | MLH1 |  |  | 1243 | 0.102 | 0.8166 | No |
| 150 | CHEK2 |  |  | 1254 | 0.101 | 0.8177 | No |
| 151 | SRSF1 |  |  | 1315 | 0.097 | 0.8132 | No |
| 152 | POLE |  |  | 1368 | 0.094 | 0.8096 | No |
| 153 | GSPT1 |  |  | 1377 | 0.093 | 0.8107 | No |
| 154 | NUDT21 |  |  | 1379 | 0.093 | 0.8125 | No |
| 155 | POLD1 |  |  | 1452 | 0.089 | 0.8066 | No |
| 156 | CIT |  |  | 1472 | 0.088 | 0.8064 | No |
| 157 | CCP110 |  |  | 1554 | 0.084 | 0.7994 | No |
| 158 | TBRG4 |  |  | 1574 | 0.083 | 0.7991 | No |
| 159 | RNASEH2A |  |  | 1579 | 0.082 | 0.8004 | No |
| 160 | WEE1 |  |  | 1617 | 0.081 | 0.7981 | No |
| 161 | GINS3 |  |  | 1639 | 0.080 | 0.7975 | No |
| 162 | NBN |  |  | 1657 | 0.079 | 0.7973 | No |
| 163 | PRPS1 |  |  | 1696 | 0.077 | 0.7949 | No |
| 164 | DCLRE1B |  |  | 1724 | 0.076 | 0.7935 | No |
| 165 | ING3 |  |  | 1778 | 0.073 | 0.7893 | No |
| 166 | XPO1 |  |  | 1871 | 0.070 | 0.7809 | No |
| 167 | CNOT9 |  |  | 1936 | 0.067 | 0.7754 | No |
| 168 | EED |  |  | 1981 | 0.065 | 0.7720 | No |
| 169 | ZW10 |  |  | 1994 | 0.065 | 0.7720 | No |
| 170 | HUS1 |  |  | 2021 | 0.064 | 0.7706 | No |
| 171 | NUP153 |  |  | 2025 | 0.064 | 0.7716 | No |
| 172 | ASF1A |  |  | 2045 | 0.063 | 0.7708 | No |
| 173 | NUP107 |  |  | 2080 | 0.062 | 0.7685 | No |
| 174 | PRKDC |  |  | 2154 | 0.059 | 0.7618 | No |
| 175 | SMC6 |  |  | 2163 | 0.059 | 0.7622 | No |
| 176 | STAG1 |  |  | 2478 | 0.048 | 0.7292 | No |
| 177 | PDS5B |  |  | 2565 | 0.046 | 0.7209 | No |
| 178 | SUV39H1 |  |  | 2604 | 0.044 | 0.7177 | No |
| 179 | PPM1D |  |  | 2605 | 0.044 | 0.7187 | No |
| 180 | ESPL1 |  |  | 2838 | 0.038 | 0.6944 | No |
| 181 | PPP1R8 |  |  | 3338 | 0.027 | 0.6409 | No |
| 182 | TP53 |  |  | 3495 | 0.024 | 0.6246 | No |
| 183 | PNN |  |  | 3632 | 0.022 | 0.6103 | No |
| 184 | MYC |  |  | 3842 | 0.018 | 0.5881 | No |
| 185 | BRMS1L |  |  | 3863 | 0.018 | 0.5863 | No |
| 186 | CDKN1B |  |  | 4019 | 0.015 | 0.5699 | No |
| 187 | HMGA1 |  |  | 4135 | 0.013 | 0.5577 | No |
| 188 | WDR90 |  |  | 4596 | 0.006 | 0.5081 | No |
| 189 | ORC2 |  |  | 4999 | -0.000 | 0.4646 | No |
| 190 | PAN2 |  |  | 6009 | -0.017 | 0.3558 | No |
| 191 | PMS2 |  |  | 6282 | -0.021 | 0.3268 | No |
| 192 | LBR |  |  | 7064 | -0.038 | 0.2432 | No |
| 193 | TFRC |  |  | 8650 | -0.107 | 0.0740 | No |
| 194 | LUC7L3 |  |  | 8798 | -0.122 | 0.0606 | No |
| 195 | PRDX4 |  |  | 9151 | -0.178 | 0.0263 | No |
| 196 | CDKN1A |  |  | 9301 | -0.230 | 0.0150 | No |
Table: GSEA details [plain text format]

  

Fig 2: HALLMARK\_E2F\_TARGETS: Random ES distribution      
 Gene set null distribution of ES for **HALLMARK\_E2F\_TARGETS**
